# Whole-slide imaging in hematopathology: Current state, challenges and future opportunities

**DOI:** 10.1016/j.jpi.2026.100708

**Published:** 2026-08-19

**Authors:** Reyhaneh Norouziani, Shay Yakobov, Hamid R. Tizhoosh, Clinton J.V. Campbell

**Affiliations:** a Faculty of Health Sciences, McMaster University, 1280 Main Street West, Hamilton, ON L8S 4L8, Canada; b Rady Faculty of Health Sciences, Max Rady College of Medicine, University of Manitoba, 727 McDermot Avenue, University of Manitoba (Bannatyne Campus), Winnipeg, MB R3E 3P5, Canada; c KIMIA Lab, Department of Artificial Intelligence and Informatics, Mayo Clinic, Rochester, MN 55901, USA; d Division of Hematopathology, Hamilton Health Sciences, 1200 Main St. W, Hamilton, ON L8N 3Z5, Canada

**Keywords:** Whole-slide imaging, Hematopathology, Digital pathology, Artificial intelligence

## Abstract

Whole-slide imaging (WSI) is gradually being adopted for primary diagnosis in many pathology subspecialties. The benefits of WSI for the pathology workflow are numerous and include the use of artificial intelligence (AI) tools to support WSI analysis. However, digitization of hematopathology workflows has remained a challenge, in part due to the complexity of the hematopathology workflow and the unique characteristics of blood and bone marrow smears. Although AI applications to support hematopathology tasks, such as nucleated differential counts, are well-described, general implementation of these tools has been limited due to the lack of widely available WSIs. Consequently, there is an unmet need for new and innovative solutions to WSI in hematopathology. This review focuses on WSI of hematopathology cytological specimens, particularly, peripheral blood and bone marrow smears, and summarizes unique challenges and AI solutions to support WSI analysis in this area. We also discuss future opportunities for hematopathologist-driven innovation in AI and WSI in hematopathology.

## Introduction

**Fig. 1:**
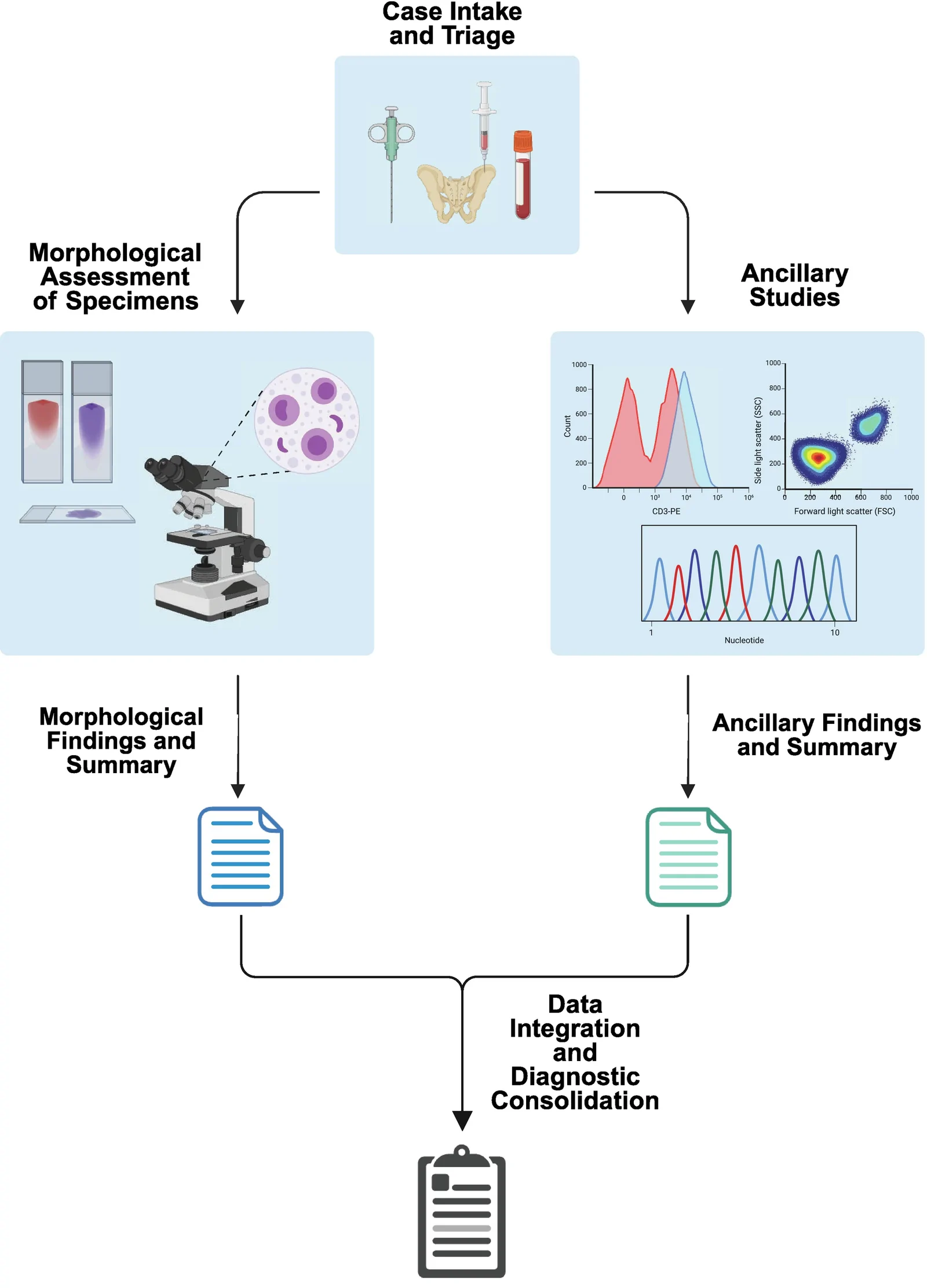
Hematopathology diagnosis represents a multistep, multimodal workflow. Following case intake and triage of specimens such as peripheral blood smear, bone marrow aspirates, or trephine core biopsies, parallel diagnostic pathways are initiated, including morphological and cytological assessment and other ancillary studies (e.g., flow cytometry, cytogenetics, and molecular assays).^6^ Each pathway generates distinct information that must undergo information integration and diagnostic consolidation by a hematopathologist. Of note, whole-slide imaging is only one component of this workflow.

Making a diagnosis in pathology is a complex multimodal task that requires the integration of information from multiple sources.^1^, ^2^ A final diagnostic interpretation entails a semantic summary of this information by an expert pathologist, which can alternatively be described as a “patient representation.”^2^ This is particularly the case in hematopathology, where inherent workflow complexity requires extraction and summarization of information from several specimen types (Fig. 1). Although viewing microscopic features of tissue (cytology or histopathology) constitutes only one part of this workflow, it is arguably the most important component of a diagnosis in pathology. A fundamental paradigm of pathology is that microscopic features of tissue are central to patient representation, as these features serve as biomarkers of health and disease.^2^ Interestingly, most of the information in the hematopathology workflow is already digital (e.g., complete blood counts (CBCs), molecular reports, flow cytometry and in some cases, histopathology whole-slide images (WSIs)).^1^ However, the digitization of peripheral blood (PB) and bone marrow aspirate (BMA) smears has lagged behind these other modalities.^1^, ^3^, ^4^ Evaluation of these smears (known as cytology) provides critical and clinically urgent information in near-real time, for example, blast counts in acute leukemias.^5^, ^6^ Therefore, there is an unmet need for reliable and efficient WSI solutions in hematopathology.

In the current context, digital pathology describes an overall diagnostic approach that includes techniques such as computational pathology and data science, machine learning/artificial intelligence (ML/AI), and WSI.^7^ Thus, WSI is only one part of digital pathology.^7^ In this review, we consider three categories for digital hematopathology imaging systems. (1) Conventional WSI: this refers to acquisition of a navigable image of the entire glass slide at diagnostic resolution that allows review of the entire specimen without restriction; (2) region of interest (ROI)-based imaging: only a portion of diagnostically relevant regions are acquired at full resolution, rather than the whole slide; and (3) computationally reconstructed imaging that generates a high resolution digital representation that may differ technically from conventional tile-based WSI acquisition. These distinctions are used throughout this review, particularly when discussing currently available platforms.

The general value propositions of WSI are well described elsewhere.^8^, ^9^ These are largely derived from WSIs of solid tissue (histopathology) specimens and include: (1) long-term institutional cost savings^10^; (2) increases in pathology workflow efficiency^10^; (3) support for remote diagnostics and peer collaboration^11^; and (4) a substrate for computational pathology techniques such as AI.^12^ Although the first two value propositions remain the subject of ongoing discussion as more experiential data are collected,^13^ the latter two generally apply to all WSI workflows.^9^, ^12^ Interestingly, the overall adoption rate of WSI remains relatively low at the time of writing, estimated at around 10%–12% of pathology labs in the USA.^14^ Of this, adoption is almost exclusive to histopathology specimens. Adoption of fully digital hematopathology workflows (i.e., including blood and bone marrow smears) is comparatively rare.^3^ General limitations to WSI across all pathology subspecialties include high upfront implementation costs and ongoing storage costs, especially for publicly funded healthcare systems and institutions with constrained financial resources.^15^ For example, real-world experience from eight European labs has shown that successful implementation of digital pathology requires a substantial investment in not only hardware such as scanners and workstations but also in software, information technology infrastructure, storage systems, and operational costs, including labor and maintenance costs.^13^ Although this study stated that none of the studied labs have digitized cytology, many of these factors are likely generalizable to hematopathology workflows. However, the specific factors that have led to the relatively delayed implementation of WSI in hematopathology remain an important and unaddressed question in the field.

**Fig. 2:**
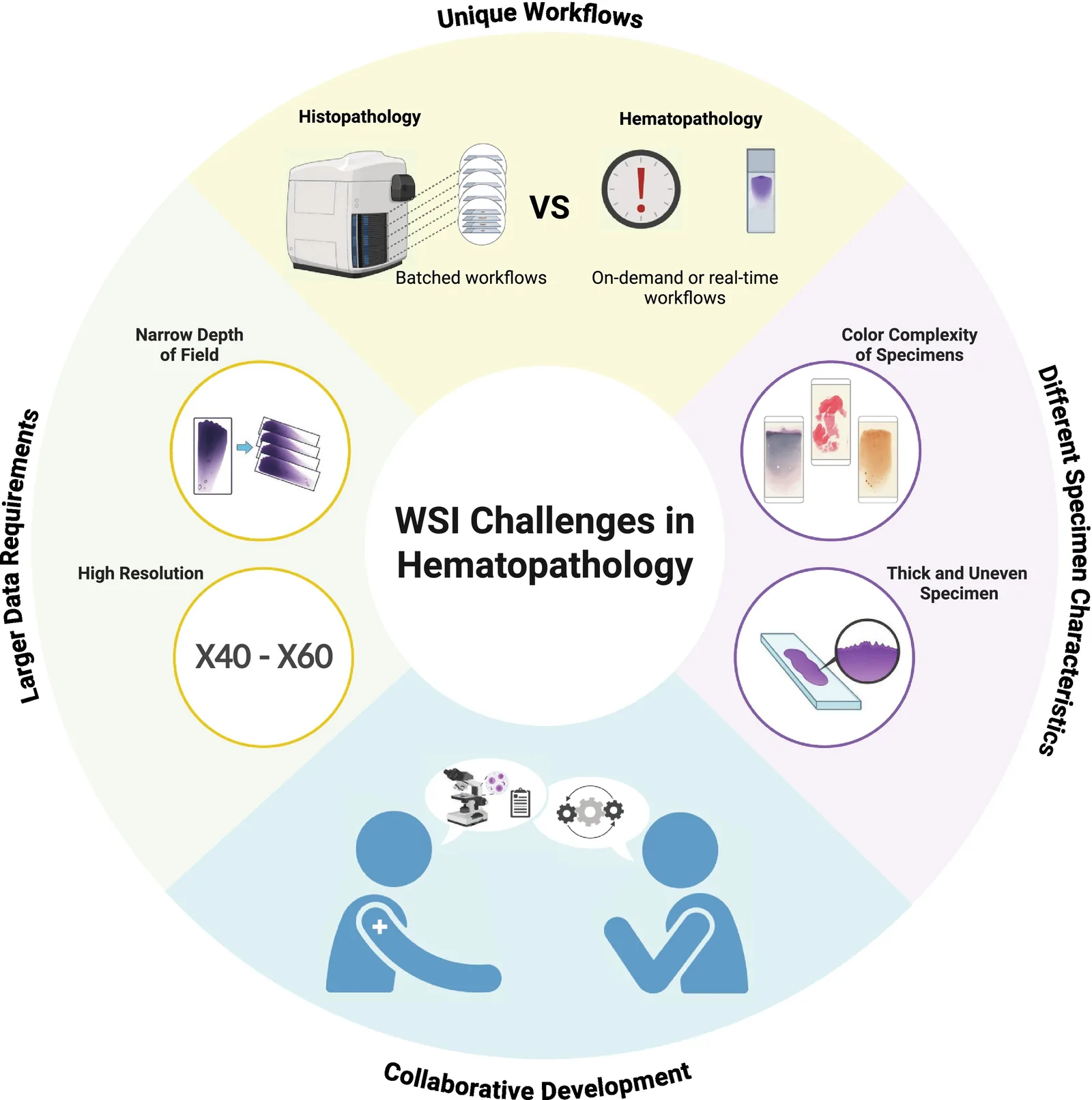
Key challenges of whole-slide imaging in hematopathology. Key challenges in hematopathology whole-slide imaging include: (1) different specimen characteristics such as variability in color, thickness, and uneven preparations,^16^ (2) larger data storage and informatics requirements due to the need for z-stacking to maintain focus; and high-resolution imaging for cytology,^4^, ^6^, ^17^ (3) unique workflows and staining requirements, as histopathology typically follows a batched workflow, whereas hematopathology involves on-demand or real-time workflow and diverse staining techniques,^6^ and (4) collaborative development that requires continuous communication between hematopathologists and vendors to improve both AI (software) and scanner (hardware) solutions.

In addition to general cost and storage limitations, there are many technical challenges inherent to the hematopathology workflow. (Fig. 2) PB and BMA smears differ fundamentally from histopathology sections, exhibiting variable thickness and heterogeneous cell distribution,^16^ making it difficult for scanners to maintain consistent focus across the entire slide.^4^, ^8^ Unlike histopathology sections, these cytology smears often cover a large portion of the slide, increasing the area that must be scanned, yet diagnostically relevant regions may occupy only small areas resulting in a low signal-to-noise ratio. High magnification (at least 40× or greater) is often required to resolve cytological details,^6^ substantially increasing file size and scanning time. The narrow depth of field at such magnifications combined with the thick and uneven nature of the specimen makes focal plane selection challenging and may require *Z*-stacking, increased storage, scanning time, and processing requirements.^8^, ^17^ Furthermore, the dense, whole-slide staining of hematopathology smears leaves minimal empty background for efficient image compression. This limits the effectiveness of storage optimization strategies used for histopathology slides stained with hematoxylin and eosin (H&E).^18^ Together, these optical, acquisition, and data-management challenges pose barriers to both the design of WSI scanners tailored for hematopathology and to routine scalable digitization of these samples.

The present review provides an overview of the current state of WSI in hematopathology. Although hematopathology includes histopathology, cytopathology, and ancillary diagnostic studies, this review focuses on the digitalization of hematopathology cytology specimens, particularly PB and BMA smears. This includes unique technical and workflow challenges of WSI of hematopathology smears, the current relevant commercial landscape, and a brief overview of AI solutions developed to support WSI of hematopathology smears. We conclude by discussing emerging opportunities for collaborative pathologist-industry innovation aimed at bridging the research-to-implementation gap.

## Review methodology

This narrative review was conducted by a literature search in PubMed and Google Scholar for studies through July 2026. The search terms included a combination of “hematopathology,” “digital pathology,” “computational pathology,” “whole slide imaging,” “bone marrow aspirate,” “peripheral blood smear,” “artificial intelligence,” “CellaVision,” “Scopio,” “Morphogo,” and “West Medica.” English language articles, peer-reviewed original research, and review articles were included, whereas case reports and conference abstracts were excluded. Additional references were tracked through relevant articles. Commercial platforms were selected and studied through peer-reviewed publications, manufacturer technical documentation, and official product websites. This review focuses on technologies and studies relevant to digital imaging in hematopathology cytology specimens, particularly PB smears and BMA smears. As this study is a narrative review, formal systematic review methodology and quality assessment were not performed.

## The hematopathology workflow

The hematopathology workflow generally includes three main components: histopathology, ancillary studies, and cytology. Histopathology involves the examination of tissue sections, such as bone marrow trephine core, lymph node, or other tissue biopsies, whereas ancillary studies include CBC data, flow cytometry, molecular diagnostics, cytogenetics, and other clinical or lab data.^6^ The hematopathologist integrates these data streams into a final consolidated diagnostic report (Fig. 1). The histopathology component in hematopathology is similar to workflows used in other pathology subspecialties and has been effectively digitized.^19^ However, cytology, which is the microscopic examination of individual cells, introduces unique challenges that remain unresolved and have contributed to the relatively slower digital transformation of hematopathology.^3^ Therefore, when we refer to digital hematopathology in the context of this review, the focus is on cytology, i.e., PB and BMA smears (which may also include bone marrow biopsy touch preparations). It should be noted that whereas evaluation of PB and BMA smears constitutes a considerable portion of the hematopathology workflow, cytological analysis of other body fluids such as cerebrospinal fluid (CSF) is also performed by hematopathologists (see below). Cytology differs from other pathology subspecialties due to its unique characteristics and technical requirements. These challenges can be broadly classified into three domains: (1) the unique characteristics of these specimens, (2) technical difficulties in the acquisition and storage of cytology WSI, and (3) the unique nature of these workflows.^6^ Together, these factors have impeded the scalability and routine clinical adoption of digital hematopathology. Each of these domains will be discussed in detail in the following sections.

### Unique attributes of hematopathology specimens

Fundamentally, every digital slide scanner consists of two key components: an optical microscope and a digital camera, which together convert analog visual information into digital information.^8^ Quality of the resulting digital image is influenced by factors in both domains. In terms of optics, important determinants include magnification, focus, and consistency of the optical path.^20^, ^21^ Regarding digitization, key factors include digital resolution, color fidelity, and image compression.^22^ These parameters impact WSI in all areas of pathology, however, become particularly limiting in hematopathology due to structural complexity, variable thickness, and high staining density of cytological specimens (Fig. 2).

Hematopathology is defined by unique specimen types that distinguish it from other pathology subspecialties. These distinct characteristics influence how WSIs are acquired, making the implementation of WSI in hematopathology fundamentally different from other pathology subspecialties. Unlike formalin-fixed, paraffin-embedded H&E-stained tissue sections, which are typically relatively thin and uniform, PB and BMA smears exhibit variable thickness, heterogeneous cell distribution, and high visual complexity.^16^ Also in contrast to H&E tissue sections, only select regions of PB and BMA smears are diagnostically relevant, with the rest contributing minimal diagnostic value while complicating image acquisition (i.e., low signal-to-noise ratio).^6^ Conversely, in H&E tissue sections, generally the entire specimen has diagnostic relevance, i.e., high signal-to-noise ratio. Furthermore, the thickness of these smears is often uneven. When coverslipped, they tend to trap air or bend, which causes light to pass unevenly through the slide. Irregularities in light path can result in localized areas of blurring or loss of focus (increased light refraction),^23^, ^24^ particularly when using higher magnifications as is common in hematopathology.^6^ Although immersion oil can help standardize the refractive index and improve optical clarity,^24^ many existing digital scanners are not designed for full-slide oil immersion.^25^ As discussed below, these features impact multiple aspects of the hematopathology WSI workflow.

These challenges are intensified by the need for higher magnification in hematopathology.^6^ Whereas most histopathology slides are routinely scanned at 20× or 40×,^26^ hematopathology smears often require 40× to 60× magnification, typically with higher numerical aperture (NA) objectives to resolve fine cytological details.^6^ As a consequence, this contributes to an increased file size of hematopathology WSIs. In addition, increasing NA inherently reduces the depth of field (the vertical range in which the image remains sharply focused) making the selection of a single focal plane challenging.^21^ In digital imaging, focal plane refers to the specific depth within the specimen at which the scanner captures the image. In thick or uneven smears, diagnostically relevant areas may be located above or below this plane. As a result, important visual details can be lost if they fall outside the narrow focus zone.^4^ One approach to address this limitation is *Z*-stacking, where multiple images are captured at different focal depths along the Z-axis to better represent the three-dimensional structure of the specimen. Although Z-stacking can improve WSI quality, it comes with significant computational trade-offs, including increased scanning time, larger file sizes, and higher storage requirements^4^, ^8^ as detailed below.

### WSI acquisition and data storage in hematopathology

Two related but distinct fundamental parameters in digital imaging are magnification and resolution. Magnification generally refers to the enlargement of the image relative to the actual object, whereas resolution generally defines the amount of fine detail that can be distinguished. In microscopy, optical resolution depends on the Rayleigh criterion, which takes into account both the wavelength of light and the NA of the objective lens.^20^:

$$R=\frac{0.61\lambda }{\mathrm{NA}}$$

Where R is the minimum resolvable distance, λ is the wavelength of the illuminating light, and NA is the numerical aperture of the objective lens. In digital pathology, resolution is typically expressed in microns per pixel, indicating the physical area on the slide that each pixel represents. According to the Nyquist sampling theorem, digital resolution should ideally be at least twice the optical resolution to sufficiently preserve visual detail.^20^ This distinction becomes particularly important in hematopathology, where higher magnifications are often necessary to resolve subtle cytological features.^6^, ^27^ As magnification increases, maintaining adequate digital resolution requires a corresponding increase in pixel density. In WSI, this means that a smaller tissue area is projected across the camera sensor, allowing the system to capture more pixels per unit area.^28^ Each pixel then represents a smaller region of the specimen, allowing the capture of finer spatial detail. For example, scanning at 40× typically yields a resolution of 0.25 μm/pixel, compared to 0.5 μm/pixel at 20×.^25^ Whereas this results in a twice the linear resolution, the total number of pixels increases by a factor of four, because both horizontal and vertical sampling densities double. This increase in sampling density allows for more precise visualization of cellular morphology. As mentioned, this is important in hematopathology, where diagnostic features may be small and sparsely distributed, e.g., Auer rods or granules in blasts. However, the benefits of higher resolution come with important computational trade-offs; for example, scanning a typical 15 mm × 20 mm specimen at 20× can generate an uncompressed file of about 3–4 GB.^29^ In comparison, scanning the same slide at 40× may yield nearly 14–15 GB, which, after JPEG2000 compression, can be reduced to roughly 500–1000 MB. However, compression in hematology WSIs is more challenging and unlikely to be as efficient, as described below.

In addition to the demands of high resolution, hematopathology also presents challenges related to depth of focus that may necessitate the use of *Z*-stacking in WSIs. The impact of magnification and *Z*-stacking on scanning time and storage requirements has been quantitatively demonstrated in a pilot study of cervicovaginal cytology slides by Wright et al. (2013).^17^ In this study, scanning a ThinPrep slide at 20× required approximately 6 min and produced a file size of around 694 MB. At 40×, the scanning time increased to approximately 15 min, with a resulting file size of 3.4 GB. When *Z*-stacking was applied, these values increased substantially: a 3-layer Z-stack required approximately 40 min and produced a file size of 10.5 GB, whereas a 7-layer *Z*-stack extended the scan time to nearly 96 min with a file size of 24 GB. Although these measurements were obtained from cervicovaginal cytology rather than hematopathology specimens, they illustrate the substantial impact of increased magnification and *Z*-stacking on scanning time and storage requirements in cytology WSI.

Beyond magnification and *Z*-stacking, the staining protocols and color complexity of hematopathology specimens introduce additional challenges for digital storage, particularly in regard to WSI compression.^30^ Data compression can be performed in two main ways: lossless compression, which preserves all original information so that no data are lost after decompression; and lossy compression, which achieves higher compression ratios by permanently discarding data.^18^ Lossless methods are preferred in medical imaging because they eliminate the risk of loss of diagnostic information. However, lossy approaches such as JPEG^31^ and JPEG 2000^32^ can achieve substantially greater size reductions and have been used in clinical WSI workflows.^33^ The choice of file format and default compression parameters also varies by vendor and can independently affect final image size, even when the same underlying compression algorithm (e.g., JPEG or JPEG 2000) is used. Although uncompressed digital images (including WSIs) generally have similar file sizes when scanned at the same magnification, the degree of compression that can be achieved can vary significantly. In WSI, the efficiency of compression of digital images depends on the color complexity of each pixel^30^ and the proportion of diagnostically relevant tissue to the “empty” background space. For instance, with JPEG 2000 compression for WSIs (JP2-WSI), the compression ratio increases roughly linearly with the ratio of empty slide to tissue area.^18^ H&E slides often contain substantial background regions (i.e., regions without tissue present), thereby allowing compression algorithms to apply higher compression to non-informative areas while preserving detail in diagnostically relevant ROI. However, this strategy is less effective for hematopathology WSIs. In Romanowsky-stained specimens, diagnostically important content (particles and cells) typically spans the entire WSI, leaving little background available for compression. As a result, these slides are inherently more difficult to compress efficiently without risking loss of diagnostic information. Although this difference may seem insignificant on a per-slide basis, its impact is likely to become substantial when used at scale in clinical settings. For example, when the number of slides is large, increased file sizes directly affect storage infrastructure and costs. Several studies have examined the storage requirements and associated expenses of WSI, identifying them as important barriers to widespread adoption.^15^ Incorporating hematopathology slides into existing digital pathology workflows will likely require substantially greater data storage capacity compared to histopathology slides.

### Differences in hematopathology workflows

Nearly all published studies and implementations of WSI today describe the use of histopathology specimens.^8^ Hematopathology workflows, particularly cytology, are distinct from histopathology and therefore warrant unique hardware solutions. In anatomical pathology, WSI normally requires batched workflows that are integrated logistically and temporally into the histopathology lab. Specimens from multiple different pathology subspecialties may be processed, stained, and scanned simultaneously.^34^ Hematopathology workflows are significantly different. For example, PB and BMA smears are frequently of high clinical urgency, and the time from patient to hematopathologist review of smears is often within 4–6 h.^6^ This almost certainly will require completely separate hardware and workflow setups from histopathology to ensure timely digitization and review of hematopathology WSIs. This is compounded by the technical limitations of hematopathology discussed above, particularly the scanning time, which is longer than for histopathology WSIs. Collectively, these workflow factors are likely to require the development of separate standalone workflows and hardware for hematopathology entire slide images.

For the practical implementation of digital hematopathology, careful integration into existing hematopathology lab workflows will be required. Many hematopathology labs already use automated analyzers for CBCs and digital morphology (DM) review by technologists. Therefore, WSI scanners will require consideration of lab and workflow setups, as well as integration with existing lab information systems and hematology analyzers. This trend is reflected by collaborations between hematology analyzer manufacturers and digital imaging companies, such as the partnership between Beckman Coulter and Scopio Labs, which aims to integrate DM solutions into existing hematology workflows.^35^ These factors should also be considered during capital equipment requests for proposals and strategically incorporated into long-term lab budgets and planning.

AI-assisted workflows also have the potential to complement the existing workflow. As summarized in Table 1, current DM analyzers, such as CellaVision, already use AI-assisted pre-classification of PB cells, with final review, verification, and correction performed by a medical lab technologist.^36^, ^37^, ^38^, ^39^ The role of AI in hematopathology is further discussed below.

**Table 1:** Comparison of commercial digital imaging platforms for hematopathology.

Platform	Specimen support	Imaging approach	Navigability	Interoperability	AI functionality	Regulatory status	Reference(s)
CellaVision DM1200/DM9600	PB; body fluids (CSF, serous, synovial)	Field/cell-selection-based; captures fixed field count, not full slide. No WSI generated.	Pre-selected fields only; no full-slide pan/zoom.	Stand-alone systems. Bi-directional LIS support (ASTM); multiple DM analyzers can share a database.	• **Peripheral blood App:** 17-class WBC differential, RBC overview, platelet estimation, non-WBC pre-classification • **Advanced RBC App:** Automated characterization of 21 RBC morphology features • **Body fluid App:** 6-class WBC pre-classification	FDA-cleared. CE-marked.	36, 37, 38, 39, 59
CellaVision DI-60	PB; body fluids. Not applicable to BMA.	Field/cell-selection based; captures fixed field count, not full slide. No WSI generated.	Pre-selected fields only; no full-slide pan/zoom.	Integrates directly with Sysmex XN-series hematology analyzers.	• **Base App:** 11-class WBC pre-classification, non-WBC detection, and RBC morphology screening • **Advanced RBC App:** Automated characterization of 15 RBC morphology features • **Body fluid App:** 5-class WBC pre-classification	FDA-cleared; CE-marked.	39, 59, 60
CellaVision DC-1	PB; BMA	Field/cell-selection at 100×; automatic differential-area selection, not full-slide scan.	Review limited to auto-selected differential areas; remote review via BMA review software.	Distributed via Sysmex; BMA results savable as PDF or sent to LIS; supports secure remote access.	• **BMA App:** AI-assisted pre-classification of 16 differential classes and 3 non-differential categories • Manual reclassification of cell classes • Automated dysplastic-cell percentage and M:E ratio • **Peripheral blood App:** Same functionality as DM1200/DM9600 • Does not support advanced RBC or body fluid Apps	BMA App CE-marked: Not FDA-cleared at time of writing	38, 61, 62
Scopio X100/X100HT	PB; BMA	“Full-Field” (FF) imaging: monolayer + feathered edge at up to 100× across large scan area.	Browser-based real-time remote review/collaboration via secure hospital network (manufacturer materials)	DICOM integration; LIS integration	• **FF-PBS:** AI-assisted 200-cell WBC differential (14 classes), platelet localization, and platelet estimation • **FF-BMA:** AI-assisted 500-cell differential with pre-classification of nucleated cells into 16 categories • Specimen quality assessment	FDA-cleared: CE-marked;	51, 54, 63
Morphogo	BMA	WSI (40× full-slide scan, 100× ROI review)	Multiple slide navigation modes	Unknown	• Classification of >40 nucleated cell types • Specimen quality assessment	Not FDA-cleared at time of writing, Registered in China, Japan, Europe, UK, and Australia	^64^
West Medica Vision A1/Vision Assist/Vision Pro/Vision Ultimate	PB; body fluids (CSF, exudates); BMA; reticulocytes.	ROI-based: automatic 10× pre-scan for cellularity/ROI detection, then 100×/1000× capture within selected ROIs only.	Manual inclusion/exclusion of fields from ROI Manager; full-slide “cells-in-view” navigation for body fluids; remote review via Vision Remote	Bi-directional LIS (LIS2-A2/ASTM, HL7), Ethernet.	• **Vision Hema:** 12-class WBC differential • **Vision extended RBC:** 26-parameter RBC morphology classification • **Vision extended PLT:** 4-category platelet classification • **Vision RET:** Automated reticulocyte detection with quantitative indices • **Vision bone marrow:** 10-class bone marrow cell classification, automatic megakaryocyte detection • **Vision body fluids:** 10-category classification	CE-marked; Not FDA-cleared at time of writing	65, 66, 67

### Quality assurance considerations

Assessment of specimen adequacy is an important component of workflow quality assurance (QA) in digital pathology and should be considered during the implementation of WSI workflows.^40^ Specimen adequacy may be assessed before scanning as part of routine lab quality control, during the initial stage of scanning using low-resolution preview images, or after scanning through evaluation of the digital image. Because WSI is resource-intensive in terms of scanning time, storage, and computational requirements, assessing specimen adequacy before or during the initial stage of scanning may reduce unnecessary resource utilization. Additionally, AI-based image quality assessment algorithms applied to low-resolution preview images could assist in automatically identifying inadequate specimens or artifacts before high-resolution image acquisition, although such approaches require further validation before routine clinical implementation.^40^ Whereas such approaches have been described in WSI of histopathology slides,^41^, ^42^ their use in hematopathology remains largely unknown and will likely require further validation in hematopathology specimens.

### Human factors and adoption of whole-slide imaging in hematopathology

Beyond these technical challenges, workflow efficiency and pathologist preferences may also influence the adoption of WSI by pathologist.^8^ Particularly in hematopathology, it is important to note that a glass PB smear or even a BMA smear can be reviewed and interpreted by an experienced hematopathologist in seconds to minutes with exquisite optical detail and resolution by using conventional light microscopy. Any viable and accepted WSI technology in hematopathology will not only need to be temporally and visually non-inferior but also have inherent value-add such as AI or WSI analysis support systems to be successfully adopted in clinical practice.

## Current commercial landscape in hematopathology whole-slide imaging

Most existing systems supporting WSI in hematopathology support digitization of PB smears, whereas there are fewer options for BMA smears. Compared with BMA smears, the digital analysis of PB smears is more established, with several commercial platforms integrated into routine lab workflows. Among the available PB smear analyzers, the market is dominated by a single vendor, CellaVision.^43^ CellaVision's DM analyzers are widely adopted in hematology workflows.^44^ These instruments automatically identify the monolayer region of a PB smear capturing high-magnification images (typically 50× for red blood cells and 100× oil immersion for white blood cells), and use AI-based algorithms to pre-classify white blood cells into their respective categories.^44^, ^45^ This process generates an automated preliminary PB smear leukocyte differential count. Classification results are reviewed and often corrected by a skilled morphologist before final reporting (known as a manual differential count, or smear review by a medical lab technologist).^44^ As of very recently, CellaVision also offers a BMA application that the manufacturer states allows AI-assisted pre-classification and review of hematopoietic precursors in the aspiration.^46^ However, similar to its PB smear workflow, the system captures selected cells or fields rather than generating a conventional WSI. As such, it is more appropriately classified as a ROI-based imaging system rather than a conventional WSI platform. It should also be mentioned that there are currently some commercially available analyzers that can perform cell differential counting on other specimens analyzed by hematopathologists, such as CSF.^47^ However, digital scanning and generation of WSIs from these samples share many of the same technical challenges that are inherent in all cytology digital scanners.

There are at least three commercial platforms that claim to provide dedicated solutions for digitizing BMA smears: Scopio Labs,^48^ Morphogo,^49^ and West Medica.^50^ Scopio Labs has introduced the X100 and X100HT systems, both reported to be capable of capturing “full-field (FF)” images of BMA smears at 100× oil immersion magnification.^51^ According to the classification adopted in this review, the Scopio systems are not conventional WSI platforms but instead employ a computationally reconstructed imaging approach. This classification is based on Scopio's description of its FF technology as a digital AI-augmented approach that simultaneously preserves the low resolution slide and 100× single-cell detail.^52^ Although Scopio states that it uses a physics-based reconstruction approach to generate 100 × −resolution FF images, the company does not provide technical details regarding the specific methods or algorithms underlying this reconstruction.^52^ The X100 requires manual application of immersion oil and coverslipping, whereas the X100HT automates these steps.^51^ Both systems are FDA-cleared and work in combination with Scopio's FF-BMA AI software module, which is also FDA-cleared.^53^ The FF-BMA application claims to support automated 500-cell nucleated differential counts (NDCs), classification of hematopoietic cells into 16 categories and provides users with the ability to confirm or adjust the differential as needed.^54^ The second system, Morphogo, is classified as a ROI-based imaging platform according to the framework adopted in this review, as it performs an initial whole slide scan at 40× magnification, followed by automated scanning of a detected ROI at 100× oil immersion to locate nucleated cells.^55^ This workflow reflects the need for higher-resolution imaging of diagnostically relevant cells than is provided by the initial 40× overview scan. Morphogo has received regulatory approval in China, Japan, Europe, Australia, and the United Kingdom, and has been evaluated in several clinical studies.^56^ The third platform, West Medica's Vision Bone Marrow series (Assist, Pro, and Ultimate), claims to support automatic scanning with a range of magnifications including 10×, 50× oil, 60× oil, and 100× oil.^50^ Higher-end models incorporate features such as automated oil dispensers. The manufacturer states the accompanying Vision Bone Marrow software facilitates AI-assisted cell pre-classification, differential counting, and reporting.^27^

An overview of currently available commercial digital systems relevant to hematopathology workflows is summarized in Table 1. In general, all of these platforms contain proprietary imaging technologies, leaving hematopathologists and other users without a complete understanding of how digital images are generated. This may impede the larger adoption and acceptance of these technologies by hematopathologists. However, several studies have reported substantial concordance between AI-assisted digital differential counts generated by platforms such as Scopio Labs^57^, ^58^ and Morphogo^55^ and conventional expert manual microscopy review. Regardless, additional independent, multicenter, and prospective validation studies are likely necessary to establish their broader clinical utility.

## Applications of artificial intelligence in hematopathology

The application of AI in pathology is closely related to the digitization of pathology workflows. In pathology, the use of AI entails the use of computational software tools that support the interpretation or analysis of WSIs.^12^ Most proposed AI tools to date are based on deep learning architectures, including transformer architectures.^68^ Digitalization enables the creation of WSI datasets, which serve as the foundation or substrate for the development of AI algorithms. However, in hematopathology, the adoption of WSI remains limited, and as a result, AI applications in this field are still in their early stages.^68^ At present, we could not locate open-access datasets of hematopathology WSIs. There are collections of individual cell or region-level images; these lack the size and context needed to develop more advanced AI models, such as those now common in histopathology.^69^, ^70^, ^71^ Continued advancement of dedicated WSI scanners for hematopathology combined with the release of public datasets will be required to accelerate AI development in this domain.

Despite these challenges, researchers are exploring AI applications for a range of tasks in hematopathology.^1^, ^2^, ^5^, ^19^, ^55^, ^68^, ^72^, ^73^ Automated NDCs represent a leading example. For instance, Tayebi et al. proposed one of the first end-to-end solutions for automated bone marrow cytology, including an augmented NDC known as a histogram-of-cell-types.^5^ Similarly, Scopio Labs' FF-BMA AI application performs a 500-cell NDC,^51^, ^53^ and their FF PB smear AI-based cell classification system that was assessed in a multicenter study, demonstrating acceptable agreement with conventional manual review.^57^ AI solutions have also been proposed for automated ROI detection,^5^ an essential step toward fully automated cell detection. Within ROI-based imaging workflows, this capability has the potential to enable higher magnification scanning of diagnostically important regions of slides without prohibitive time and storage burdens, as it may reduce the area scanned at high resolution. In addition, disease detection and classification models have been developed to identify morphological patterns consistent with conditions such as acute leukemias or myelodysplastic neoplasms to support triage and accelerate review.^74^ Whereas many of these approaches remain investigational, several AI-assisted digital hematopathology systems have received FDA clearance for clinical use, as summarized in Table 2.

**Table 2:** FDA-authorized AI-assisted digital hematopathology systems.

AI module	Regulatory status	Intended use	Validation design	Population	Performance	Reference(s)
CellaVision DM96 Automatic Hematology Analyzer	FDA-cleared (510(k) K033840)	Automated cell-locating device that locates and presents images of peripheral blood cells; trained operators verify or modify the suggested classification	Clinical comparison with the DiffMaster Octavia predicate device following NCCLS H20-A methodology; complementary testing for cell location, precision, and overview image quality.	Not reported	Met predefined performance requirements; demonstrated equivalent accuracy, precision, clinical sensitivity, and specificity to the predicate device.	82, 83, 84
CellaVision Advanced RBC Application	FDA-cleared (510(k) K171315)	Automated cell locating with operator verification of suggested cell classifications	Five-site method-comparison study against manual microscopy and automated differential cell counters, plus repeatability and reproducibility testing across three clinical labs.	1598 blood samples from pediatric and adult patients (up to 101 years), including healthy individuals and patients with hematological disorders	Color (87.7% efficiency, 82.1% sensitivity, 90.5% specificity); shape (88.5%, 86.6%, 89.9%); inclusions (94.4%, 87.1%, 95.3%); size (81.3% overall agreement,80.0% positive percent agreement (PPA), 82.9% negative percent agreement (NPA))	^85^
CellaVision DM96 Body Fluid Application	FDA-cleared (510(k) K080595)	Automated cell locating for differential white blood cell counts on cytocentrifuged body fluid preparations, with operator verification of suggested cell classifications.	Two-site method-comparison study against manual microscopy with precision/reproducibility evaluation	156 body fluid samples (89 CSF, 24 peritoneal fluid, 46 pleural fluid).	Regression-based agreement with manual microscopy (R^2^ = 0.9566–0.9903 across reported cell classes); short-term imprecision equivalent to the reference method	^86^
CellaVision DM1200 Body Fluid Application	FDA-cleared (510(k) K102778)	Automated cell locating for differential white blood cell counts on cytocentrifuged body fluid preparations, with operator verification of suggested cell classifications.	Two-site method-comparison study using the CellaVision DM96 Body Fluid Application as the predicate method, with repeatability testing and three-site reproducibility testing.	260 body fluid samples (62 CSF, 151 serous fluid, 47 synovial fluid).	Regression-based agreement with the predicate method (R^2^ = 0.9273–0.9932 across reported cell classes); repeatability and reproducibility met predefined acceptance criteria.	^87^
Peripheral Blood Application (DM1200/ DI-60)	FDA-cleared (510(k) K092868)	Automated cell locating, presentation and classification of peripheral blood smear images with operator verification of suggested cell classifications.	Clinical evaluation comparing the DM1200 with the predicate device (DM96) according to CLSI H20-A2 and CLSI EP05-A2, with complementary cell-location testing (study details not reported).	Not reported	Accuracy, precision, clinical sensitivity, and specificity fulfilled predefined requirements (quantitative performance metrics not reported)	88, 89, 90
X100 with Full Field Peripheral Blood Smear (PBS) Application	FDA-cleared (510(k) K201301)	Automated cell locating and display of WBCs, RBCs, and platelets to assist qualified technologists with WBC differential, RBC morphology evaluation, and platelet estimation.	Three-site method-comparison study against manual light microscopy, with repeatability and reproducibility studies.	645 peripheral blood specimens: 335 from healthy subjects and 310 from subjects with specific disease conditions	WBC Deming-regression results met predefined acceptance criteria; overall WBC differential efficiency 96.29%, sensitivity 87.86%, and specificity 97.62%. RBC morphology overall agreement was 99.77%. Platelet-estimation efficiency was 94.89%, sensitivity 90.00%, and specificity 96.28%. Repeatability and reproducibility met predefined acceptance criteria.	^91^
X100 /X100HT Full Field Bone Marrow Aspirate (BMA) Application)	FDA De Novo authorized (DEN230034)	Automated cell locating and image presentation of Romanowsky-stained BMA smears to assist trained operators with bone marrow smear-quality assessment and blast, plasma-cell, and M:E-ratio estimation; also displays Prussian Blue–stained smear images	Three-site clinical comparison vs. manual microscopy; DSS-output precision assessed via single-site repeatability and three-site reproducibility studies.	615 Romanowsky-stained BMA slides; 180 Prussian Blue–stained BMA slides;	Clinical and precision studies met predefined acceptance criteria. Accuracy vs. manual microscopy: specimen quality 98.21%; blast estimation (<5%) 89.08%, (<20) 95.41%; plasma-cell estimation 96.24%; M:E ratio—normal vs. abnormal 93.35%, normal vs. increased 86.06%, and normal vs. decreased 78.78%.	^92^

Beyond WSI analysis, natural language processing tools, including the use of large language models have been used to extract structured information from free-text hematopathology reports. For example, Mu et al. developed a transformer-based BERT model that extracts diagnostically relevant semantic embeddings from pathology synopses and maps them to semantic labels, potentially facilitating large-scale WSI dataset annotation with reduced manual labeling.^75^ More recently, AI in the medical field has begun to explore foundation models,^76^ which are large, self-supervised models trained on extensive datasets and adaptable to a variety of tasks.^77^ However, adapting such models to pathology remains challenging due to limited rates of digitization and the scarcity of large, publicly available datasets.^78^ In hematopathology specifically, these limitations are even more pronounced as discussed. Recently, a cell-level model named DinoBloom^79^ was published, trained on 380,000 images of PB and bone marrow cells. DinoBloom demonstrated strong generalization and high performance, although its scope is limited to single-cell hematology images and requires further real-world clinical validation.

Presently, AI in hematopathology remains largely in the research domain, limited by the relatively slow pace of digitization and the scarcity of large and diverse WSI datasets in hematopathology. Most published studies have been conducted using small, single-center datasets, and few have undergone independent external or multicenter validation.^80^

Furthermore, differences in specimen preparation, staining protocols, and image acquisition devices may reduce algorithm performance across institutions.^81^ Consequently, rigorous validation is necessary before clinical adoption.^40^ Regulatory and professional recommendations regarding AI validation and implementation are discussed further in Unique opportunities for pathologist-driven innovation.

## Unique opportunities for pathologist-driven innovation

Despite the challenges described, there are opportunities for pathologist-driven innovation in WSI, particularly in hematopathology. These may entail close academic-research collaboration between practicing hematopathologists and industry partners, allowing collaborative technological development. The current model for hardware and AI solutions in pathology generally entails vendors working with pathologists to validate pre-developed scanners and AI tools in clinical settings.^57^ Professional pathology organizations have published formal recommendations for validation of digital pathology systems.^93^ The College of American Pathologists first published guidelines for validating WSI for diagnostic purposes in 2013, which were updated in 2022 in collaboration with the American Society for Clinical Pathology and the Association for Pathology Informatics.^94^ However, whereas the updated guideline includes hematopathology histopathology cases, it has excluded cytology specimens, including hematopathology PB and BMA smears. More recently, the Digital Pathology Association (DPA) published recommendations addressing the validation and clinical implementation of AI-enabled digital pathology tools into clinical practice.^40^

Whereas these guidelines emphasize validation and clinical implementation, they provide relatively little guidance on how practicing pathologists can contribute during earlier stages of technology development. Consequently, pathologist involvement often occurs after key technological decisions have already been made, limiting collaborative opportunities to identify practical workflow challenges and unmet clinical needs during system design. If a pathologist cannot generally understand imaging or AI technologies, confidence in these tools may be reduced, potentially limiting their adoption in routine practice.^40^ Although regulatory approval, such as FDA clearance or approval, provides assurance that the systems meet regulatory standards in terms of safety and effectiveness, it does not necessarily provide pathologists with information regarding how the devices function or how their outputs are generated. We acknowledge that disclosure of proprietary source code or trade secrets is not realistic or feasible. However, consistent with the DPA's emphasis on transparency in AI-enabled diagnostic workflows,^40^ we suggest vendors should provide standardized documentation describing imaging methodology; use of computational reconstruction or AI-based image enhancement; and how these processes may influence differences between the original glass slide and the final digital image presented to the pathologist. Pathologists should know whether a digital image was generated using conventional WSI, ROI-based imaging, or computationally reconstructed imaging. For ROI-based imaging, any portions of the slide that have not been imaged should be clearly indicated. For computationally reconstructed imaging, vendors should disclose whether and how the image has been upsampled (i.e., its apparent resolution has been computationally increased by generating additional pixels rather than collecting new optical information) or reconstructed using AI or other computational techniques. For AI-enabled tools, vendors should disclose the general model architecture and summary information about the characteristics of the dataset used for training and validation, while preserving commercially sensitive implementation details. Often, these questions can only be addressed by viewing the original patents, which is not practical or reasonable for pathologists.

A possible schema would be to have pathologists (including hematopathologists) continuously and longitudinally engaged by vendors during technical development through open, academic–commercial partnerships. This will allow for efficient, transparent, and clinically relevant product development and accelerate implementation of WSI and AI technologies. Rather than pose a challenge, we view this as a unique opportunity for pathologist-driven innovation toward the implementation of complete slide imaging solutions. Promising areas of collaborative research include *Z*-stacking, volumetric imaging, and extended-focus imaging, which computationally reconstructs multiple focal planes into a single sharp image.^25^ Other valuable directions are AI-guided ROI acquisition for ROI-based imaging and efficient data compression, which are particularly relevant in hematopathology.^30^, ^72^ With direct input from pathologists, these tools could be customized to meet the needs of individual laboratories. Such collaborations could also lead to the development of application programming interfaces supporting locally developed and validated AI tools. It is important to recognize that these advances depend on the appropriate computational infrastructure including WSI storage and sufficient power, cooling and maintenance systems. Table 3 summerizes current linitations of WSI in hematopathology, alongside proposed innovations and their anticipated impact.

**Table 3:** Current limitations, proposed innovations, and expected impact of whole-slide imaging in hematopathology.

Current limitations	Proposed innovations	Expected
Limited conventional (histopathology-oriented) WSI solutions for peripheral blood smears and bone marrow aspirates.^27^	Scanners and optimized image acquisition protocols specifically designed for hematopathology smears.	Routine digitization of hematopathology specimens with improved workflow efficiency.
Large image sizes and high storage requirements^4^, ^95^	Advanced image compression or ROI detection algorithms in hematopathology smears; cloud and hybrid storage; scalable infrastructure	Reduced storage burden and improved data accessibility conduced to hematopathology workflows.
Limited interoperability between vendors in whole-slide imaging.^95^	Standardized image formats and greater cross-platform interoperability,^96^ which includes hematopathology.	Seamless multivendor data sharing and digitization of complex hematopathology workflows.
Limited transparency of proprietary whole-slide image acquisition and reconstruction in hematopathology.^40^	Standardized technical documentation and criteria with all whole-slide imaging technologies describing imaging methodologies used.	Greater user confidence, easier validation, and supporting broader clinical acceptance by pathologists.
High implementation costs and infrastructure requirements.^15^	More affordable hardware, cloud computing, and institutional investment; efficient and hematopathology-specific image acquisition hardware and compression techniques.	Increased adoption hematopathology whole-slide imaging across labs of various size and resources.

## Conclusion

Adoption of WSI in hematopathology has been slower than in other fields of pathology. Hematopathology is a multimodal discipline, and whereas some aspects of the workflow are digitized, the cytology component presents unique challenges to digitization.^3^ These challenges stem from the distinct characteristics of hematopathology smears and the relatively complex diagnostic workflow.^6^ The need for higher magnification, the uneven surface of specimens, and specimen staining characteristics contribute to large data volumes and lower compression efficiency. Early AI research in hematopathology is promising, including automated ROI detection,^5^ cell classification and NDCs,^5^, ^55^ and disease detection.^74^ Translating AI tools into routine hematopathology practice has been challenging due to limited high-quality annotated hematopathology WSI datasets.^34^, ^68^ Whereas AI systems often perform well in controlled settings, their closed technical designs restrict rigorous assessment and limit opportunities for adaptation to specific institutional needs.^40^ Although a limited number of commercial platforms have begun to address these issues, there is an unmet need for scanners that can fulfill the demands and unique requirements of hematopathology workflows to produce timely, reliable, and high-quality WSIs of hematopathology smears.^50^, ^51^, ^55^ Despite these challenges, it is an exciting time in the field given a high potential for collaborative technological innovation in hematopathology WSI. An overview of these challenges and opportunities are summarized in Fig. 2.

## CRediT authorship contribution statement

RN wrote and edited the manuscript. SY and HRT contributed to writing and editing the manuscript. CJVC conceived the manuscript, provided conceptual guidance, and contributed to writing the manuscript.

## Funding

Funding for this work was partially supported by an Ontario Centre of Innovation (OCI) Collaborate to Commericialize (C2C) Grant, OCI#36612 (salary support for first author).

## Declaration of generative AI and AI-assisted technologies in the manuscript preparation process

During the preparation of this work, the authors used ChatGPT (OpenAI, GPT-5) in order to refine the language and improve the clarity of this manuscript. After using this tool, the authors reviewed and edited the content as needed and take full responsibility for the content of the published article.

## Declaration of competing interest

CJVC is a member of the Scientific Advisory Board of Huron Digital Pathology, and is a lead investigator in a funded academic—research collaboration evaluating a digital hematopathology scanner manufactured by Huron Digital Pathology. The authors declare that they have no known competing financial interests or personal relationships that could have appeared to influence the work reported in this article.
